# Viscoelastic Testing in Pediatric Mechanical Circulatory Support

**DOI:** 10.3389/fmed.2022.854258

**Published:** 2022-05-06

**Authors:** Katherine Regling, Arun Saini, Katherine Cashen

**Affiliations:** ^1^Division of Hematology Oncology, Department of Pediatrics, Children's Hospital of Michigan, Central Michigan University School of Medicine, Detroit, MI, United States; ^2^Division of Critical Care Medicine, Department of Pediatrics, Texas Children's Hospital, Baylor University School of Medicine, Houston, TX, United States; ^3^Division of Critical Care Medicine, Department of Pediatrics, Duke Children's Hospital, Duke University School of Medicine, Durham, NC, United States

**Keywords:** extracorporeal membrane oxygenation, thromboelastography, thromboelastometry (ROTEM®), ventricular assist device (VAD), pediatric, child-age

## Abstract

Pediatric mechanical circulatory support can be lifesaving. However, managing anticoagulation is one of the most challenging aspects of care in patients requiring mechanical circulatory support. Effective anticoagulation is even more difficult in pediatric patients due to the smaller size of their blood vessels, increased turbulent flow, and developmental hemostasis. Recently, viscoelastic testing (VET) has been used as a qualitative measure of anticoagulation efficacy in patients receiving extracorporeal membrane oxygenation (ECMO) and ventricular assist devices (VAD). Thromboelastography (TEG®) and thromboelastometry (ROTEM®) provide a global qualitative assessment of hemostatic function from initiation of clot formation with the platelet-fibrin interaction, platelet aggregation, clot strength, and clot lysis. This review focuses on the TEG®/ROTEM® and important laboratory and patient considerations for interpretation in the ECMO and VAD population. We summarize the adult and pediatric ECMO/VAD literature regarding VET values, VET-platelet mapping, utility over standard laboratory monitoring, and association with outcome measures such as blood product utilization, bleeding, and thrombosis.

## Introduction

Mechanical circulatory support in children is provided utilizing extracorporeal membrane oxygenation (ECMO) or ventricular assist devices (VADs). These therapies are used for respiratory, cardiac, or extracorporeal cardiopulmonary resuscitation (ECPR) support. While ECMO and VADs can be lifesaving, patients are at increased risk of bleeding (due to critical illness and systemic anticoagulation) and thrombosis. According to the Extracorporeal Life Support Organization (ELSO) registry, neonatal and pediatric patients make up about one-half of overall ECMO runs (1). In the bleeding and thrombosis during ECMO (BATE) study performed prospectively at eight centers affiliated with the Collaborative Pediatric Critical Care Research Network (CPCCRN), bleeding events were observed in 70% of children and thrombotic events in 38%. The presence of either bleeding or thrombosis was associated with adverse cognitive and functional outcomes, and bleeding events were associated with increased mortality (2). The Berlin Heart EXCOR Pediatric VAD Investigational Device Exemption (IDE) study, which included children ≤ 16 years of age across 17 pediatric cardiac centers, reported major bleeding occurred in 43% of patients, neurologic events in 28%, and pump changes for suspected/confirmed thrombosis in 56% of patients (3). There were five patient deaths in this cohort and four of these were related to thrombotic events (3). While these studies are informative, the true incidence of bleeding and thrombosis in pediatric ECMO/VAD is unknown due to a limited number of prospective outcome studies, variable definitions of bleeding and thrombosis used, and a lack of validated severity of bleeding and thrombosis scoring tools. We know that bleeding and thrombosis contribute to morbidity and mortality. Attempts to mitigate these risks have focused on circuit modifications, anticoagulant medications, and changes in hemostatic monitoring including viscoelastic testing (VET).

In this review, we provide an overview of standard anticoagulant and antiplatelet medications, traditional laboratory measures of anticoagulation, a summary of thromboelastometry and thromboelastography technique, current evidence to support VET in pediatric ECMO/VAD, and limitations of VET in this population.

## Overview of Anticoagulant Medications and Platelet Inhibitors In Ecmo/Vad

The ultimate goal of anticoagulation during extracorporeal support is to prevent thrombosis that occurs with exposure of blood to the nonendothelial surface of the ECMO or VAD circuit. Activation of the alternative pathway through the complement system and inflammatory cytokines stimulates both the intrinsic and extrinsic coagulation pathways with subsequent shift in balance to a procoagulant state in the circuit (4). With this in mind, the ideal anticoagulant would target inflammatory markers, platelet activation in addition to coagulant proteins without increasing bleeding risk. Unfortunately, today no such therapeutic option exists.

### Anticoagulant Medications

Unfractionated heparin (UFH) remains the most widely used anticoagulant for neonatal and pediatric mechanical circulatory devices, specifically ECMO (5). UFH is a sulfated polysaccharide that produces its anticoagulant effect by inactivating free thrombin and activated factor X (FXa) by binding to antithrombin (AT), which induce a confirmational change in AT structure and enhance its serine protease inhibiting activity by 500–1000 folds. Heparin binds to AT by a high-affinity pentasaccharide, which is present on about a third of heparin molecules. Thrombin inhibition is dependent upon heparin binding to both the coagulation enzyme and AT; whereas inhibition of FXa is dependent only upon binding to AT (6). Thrombin bound to fibrin or subendothelium is not inhibited by the AT-UFH complex. UFH also has the ability to bind to plasma proteins, platelet factor 4, high molecular weight von Willebrand factor (vWF) and endothelial cells independently of AT which results in a variable response to heparin and even heparin resistance (6). In addition, these proteins are released during increased inflammation and activation of the coagulation system, leading to inefficient anticoagulant activity. The possibility of heparin induced thrombocytopenia (HIT) as well as wide interpatient variability are major shortcomings of UFH. Despite this, the use of UFH is well-known to treating clinicians with a short half-life and easy reversibility. Therapeutic efficacy of UFH can be measured by the activated clotting time (ACT), activated partial thromboplastin time (aPTT), anti-factor Xa activity (anti-FXa) and VET.

AT is a serine protease inhibitor necessary for heparin to exert its anticoagulant effect. Some centers include routine monitoring of AT and replacement with either fresh frozen plasma or AT concentrates. The rationale for the replacement of AT is to improve the effectiveness of heparin and reduce thrombotic complications. However, target AT levels remain unclear and guidelines for replacement with fresh frozen plasma (FFP) or AT concentrates vary significantly across centers. AT use remains controversial, as there is the potential risk of bleeding and added cost. A Cochrane review of 20 trials using AT replacement in critically ill patients showed no significant effect on mortality in all subgroup populations, including children, but had a 1.5-fold increased risk of bleeding (7). Until larger clinical trials can establish the effectiveness of AT replacement in pediatric ECMO/VAD, AT concentrates should be used with caution.

The use of direct thrombin inhibitors (DTIs) is becoming increasingly more common in the ECMO/VAD population, even in non-HIT scenarios. DTIs inhibit thrombin irrespective of whether the thrombin is free, or fibrin bound. Bivalirudin is the most commonly utilized direct thrombin inhibitor in ECMO and VADs. Bivalirudin is eliminated primarily by a non-organ mechanism (proteolysis) with only a minor (20%) component of renal clearance. Bivalirudin has the shortest half-life (about 25 min) and also has an intermediate affinity to thrombin in comparison to lepirudin (highest affinity) and argatroban (lowest affinity) (8).

Though studies are limited, bivalirudin thus far has been used effectively in both pediatric ECMO and VAD patients. In a single-center retrospective cohort, Seelhammer et al. evaluated a total of 422 ECMO patients (89 pediatric) and did not find an increase in adverse outcomes in the pediatric population. In the adult subgroup, bivalirudin was associated with improved overall survival (9). There is no consensus on bolus or continuous infusion dosing of bivalirudin. Loading dose has been reported as 0.4–0.5 mg/kg and continuous infusion rates from 0.05 to 0.5 mg/kg/h (10–12) in ECMO patients, with higher dosing reported in VADs (13). Pediatric studies are limited with mostly retrospective single-center data or small case series in the ECMO literature. Some pediatric programs have reported using bivalirudin as a first line agent with good outcomes; however, high-quality studies are lacking (11, 13–15). In the Berlin Heart EXCOR and Advanced Cardiac Therapies Improving Outcome Network (ACTION) post-approval surveillance study report, 92% of patients in the post surveillance group (post-market approval of EXCOR VAD) were transitioned to bivalirudin and also had decreased bleeding events, improved survival, lower stroke, and fewer thrombotic events (16).

### Antiplatelet Agents

Antiplatelet agents are infrequently used during pediatric ECMO. The non-biologic surface of the ECMO circuit induces ongoing platelet activation and adhesion, which leads to platelet dysfunction (17, 18). Initiation of ECMO is associated with a decline in platelet count in neonates and pediatric patients. In a large multicenter study, the unadjusted association of average daily platelet count with mortality had a roughly linear association with the log odds of mortality up to a platelet count of approximately 115 × 10^9^/L. On multivariable analyses, average daily platelet count was not independently associated with mortality however platelet transfusion volume was associated with mortality. This suggests that the platelet count was not the reason for increased mortality but factors associated with platelet transfusion (19). There is a paucity of data about adjunct antiplatelet therapy in pediatric ECMO patients with hypercoagulable states. Recently, two hypercoagulable COVID-19 adults were supported with venovenous (VV) ECMO and thromboelastography-guided antiplatelet therapy was initiated with good outcomes (20). In a case series of three pediatric patients with the multisystem inflammatory syndrome in children (MIS-C) supported with venoarterial (VA) ECMO, aspirin was used pre-ECMO cannulation due to coronary dilation. Interestingly, the only patient not treated with aspirin pre-cannulation had early circuit thrombosis requiring circuit change despite similar elevation in inflammatory markers in all patients (21). However, the Extracorporeal Life Support Organization (ELSO) registry data suggests similar frequency of thrombotic events in adult COVID-19 ECMO patients compared to the overall ELSO registry (22). Thus, there is no evidence to support routine use of antiplatelet medications in ECMO, however they may be beneficial in select cases.

Antiplatelet agents have been routinely used in the VAD population. The Edmonton anticoagulation and platelet inhibition protocol was the first widely utilized detailed guideline for pediatric VADs and included treatment with aspirin and clopidogrel (23). Antiplatelet therapy was guided by TEG®-platelet mapping. A more aggressive triple weight-based antiplatelet strategy was employed by Rosenthal et al. and this group reported 84% lower rate of stroke then the Edmonton guideline (24). With the more widespread use of DTIs for pediatric VAD patients, aspirin and dipyridamole are the most commonly used anti-platelet agents (3, 25, 26). A recent report using data from 10 centers affiliated with ACTION, showed lower stroke and major late bleeding in patients anticoagulated with DTIs. In this study, 88% of patients were also treated with antiplatelet agents, but dual antiplatelet therapy was used in less than a third of patients (27).

## Traditional Laboratory Anticoagulant Monitoring In Ecmo/Vad

The assays used and monitoring frequency varies significantly between institutions and range from once daily to multiple time points in 24 h. In addition to the assays discussed below, hemoglobin/hematocrit, platelet count and fibrinogen levels are routinely screened. Transfusion thresholds also vary by institution and by the patient's clinical status. In general, hemostatic monitoring practice has moved from ACT only to multi-assay based monitoring (28).

### Activated Clotting Time

The ACT is a whole blood, point-of-care test that measures the time it takes for clot formation using an intrinsic pathway activator. Advantageous to pediatrics, this point-of-care test is easy to perform with fast results and requires only a small amount of blood. Initially, this test was used during cardiopulmonary bypass where heparin dosing is much higher than what is used during ECMO. During ECMO, the dosing of heparin is lower and there has been poor correlation to the ACT (29). In conjunction, it is important to remember that the duration of ECMO is much longer (days to weeks) than that of cardiopulmonary bypass (hours) leading to a sustained hyperinflammatory state which may further interfere with anticoagulant monitoring. The ACT is also impacted by several other factors including hemodilution, hypothermia, coagulation factor deficiencies and platelet dysfunction, all of which may occur during ECMO therapy (30). Previous adult and pediatric ECMO studies have reported that the ACT had no correlation to UFH dose and was relatively insensitive to changes in UFH dose (31, 32). Despite these limitations, the ACT remains in broad use in up to 68 to 97% of institutions (5, 33). The most commonly reported and recommended goal ACT for ECMO is 180–220 s (28, 34, 35), but it is unclear if this reflects adequate anticoagulation for pediatric patients (36–38).

### Activated Partial Thromboplastin Time

The aPTT is a plasma-based assay that evaluates the intrinsic and common factor pathways (factors XII, XI, IX, X, V, II and fibrinogen). This test is used to monitor anticoagulation effects of both UFH and direct thrombin inhibitors. aPTT testing is inexpensive and has the ability to detect underlying factor deficiencies (inherited or acquired), vitamin K deficiency or the presence of disseminated intravascular coagulation. However, the sensitivity of aPTT to heparin effect is both analyzer and method dependent and thus each laboratory must have their own determination of normal range. These reference ranges may change frequently due to variations in aPTT reagents between lots (39). Baseline aPTT vary significantly with age due to developmental hemostasis, and thus neonates and young children have different reference ranges than adult patients. In addition, there is an age-dependent effect of UFH on the prolongation of the aPTT (40). Generally, programs target an aPTT goal of 1.5–2.5 times baseline, which is based upon the normal range of the anti-factor Xa assay at most centers. Interestingly, there is a wide variation of reported aPTT values (in pediatric ECMO and VAD) that fall in the therapeutic window of anti-Xa (28, 40, 41). Considering developmental hemostasis in this age group and wide variation of aPTT ranges, using the aPTT for monitoring alone may be insufficient.

### Anti-factor Xa Activity

Anti-FXa is another plasma-based assay that determines the anticoagulant activity of UFH by measuring the ability of heparin-bound AT to inhibit FXa. This assay is a photometric test; thus, it is important to note that hyperbilirubinemia, elevated triglyceride and heme levels interference may cause falsely low results. In addition, AT levels will influence the assay and results may also be inaccurate if the laboratory adds exogenous AT to the specimen (28). This is particularly important in neonates and young children, as these differences are likely to be more prominent (42). The main advantage of anti-FXa is that it directly measures the heparin effect rather than heparin concentration. Therapeutic levels are considered to be 0.3–0.7 IU/ml. Specifically, in mechanical circulatory devices, anti-FXa has been shown to correlate better with heparin dosing than the ACT (31), and in one study, an increase in heparin by 10 units resulted in a reproducible increase in anti-FXa level by 0.07 IU/ml (28). Thus, anti-FXa was better correlated with heparin effect than ACT which is poorly correlated with heparin dosing (32). Unfortunately, discordance between aPTT values and anti-FXa is common in pediatric ECMO and makes interpretation of these labs challenging (31).

### Antithrombin

As previously discussed, some centers routinely monitor AT levels. AT has been reported to increase by about 1% for each ECMO day and is not related to daily volume of FFP transfusions. (28). Bembea et al. found that the median baseline AT activity for which patients received treatment was 43%, range 38–64%. AT levels were inversely correlated with the ACT, even after adjustment for heparin dose, but had a strong positive correlation to anti-FXa level (28).

### Dilute Thrombin Time

The dilute thrombin time is a plasma-based assay that is used to determine the anticoagulant activity of DTIs, like bivalirudin. Here patient plasma is diluted with human fibrinogen or normal human plasma and then clotting is initiated by the addition of thrombin; the clotting time is then measured and compared to a laboratory generated standard reference curve (43). A recent study demonstrated that the aPTT and DTT have poor correlation in samples from pediatric VAD patients (44). Because the DTT is not affected by heparin, lupus anticoagulants or vitamin-K dependent factors it may be superior for monitoring anticoagulant activity; however, studies are limited and it remains unclear if the DTT is preferred over the aPTT for monitoring (43).

## Viscoelastic Tests

**Figure 1:**
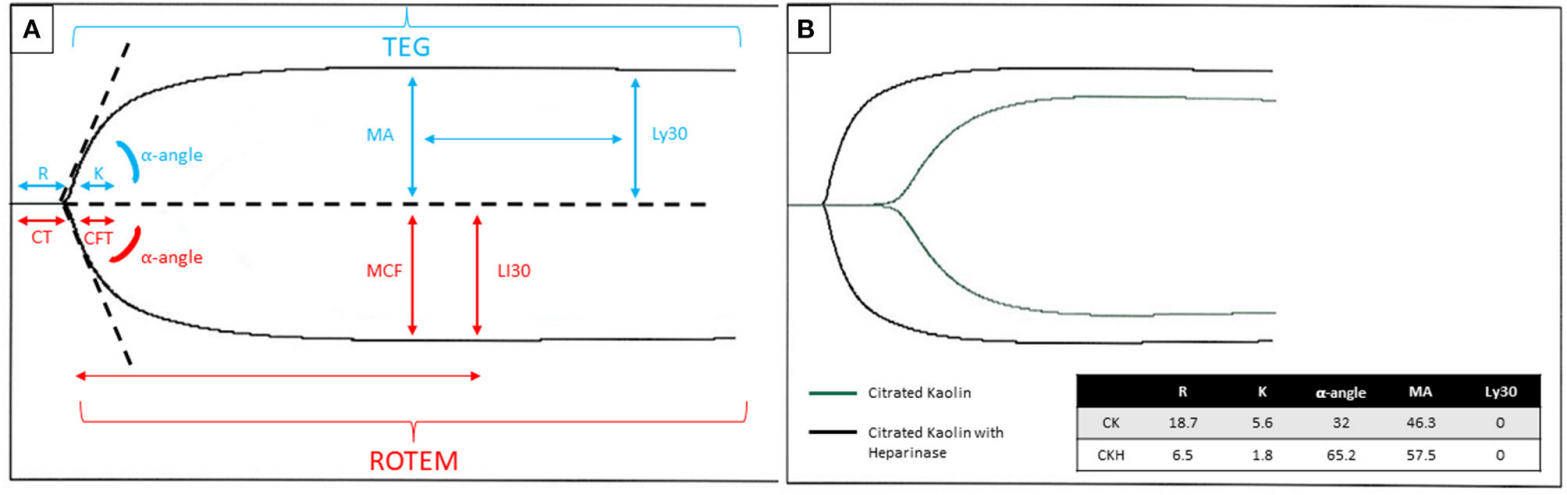
**(A)** Graphical tracing produced by the TEG® (blue font color)/ROTEM® (red font color) with parameters labeled for each respective test. The R/CT and K/CFT may be affected by heparin or other anticoagulants and coagulation factor deficiencies. The MA/MCF may be affected by fibrinogen level, absolute platelet count, or platelet dysfunction. The Ly30/LI30 may be affected by hyperfibrinolytic states or inherited/acquired factor XIII deficiency. **(B)** An example of a TEG® tracing in a patient on ECMO with citrated kaolin +/- heparinase. Legend: Blue arrow depicts the timepoint to measure Ly30, measured at 30 min after the MA. The red arrow shows the time point to measure LI30, measured at 30 min after the CT. R, reaction time, CT, clotting time; K, kinetic time; CFT, clot formation time; MA, maximum amplitude; MCF, maximum clot firmness; Ly30, lysis time 30 min after maximum amplitude; LI30, lysis time 30 min after clotting time; CK, citrated kaolin; CKH, citrated kaolin with heparinase.

Limitations in traditional laboratory testing have led some centers to adopt monitoring programs focused on multiple measures of hemostasis. Thromboelastography (TEG®) (Haemonetics Corp, Braintree, MA, USA) and thromboelastometry (ROTEM®) (Tem International GmbH, Munich, Germany) are two VETs used to assess the underlying hemostatic potential from initial clot formation through fibrinolysis in whole blood. The technology was first developed in the late 1940's and has shown benefit in monitoring coagulopathy in cardiac surgery and liver transplant (45–47). In the pediatric cardiac surgical population, VET-based transfusion algorithms for bleeding patients have led to decreased blood loss and transfusion requirements. VET is now recommended as an alternative to standard assays for managing intraoperative bleeding (48). Recently this testing has been applied to even more clinical states, including inherited coagulation disorders and in ECMO and VAD. VET can provide a more global view of hemostasis in pediatric ECMO/VAD by displaying all phases of coagulation. Figure 1 depicts the graphical tracing produced by TEG® and ROTEM®.

### VET Techniques

#### TEG®

**Figure 2:**
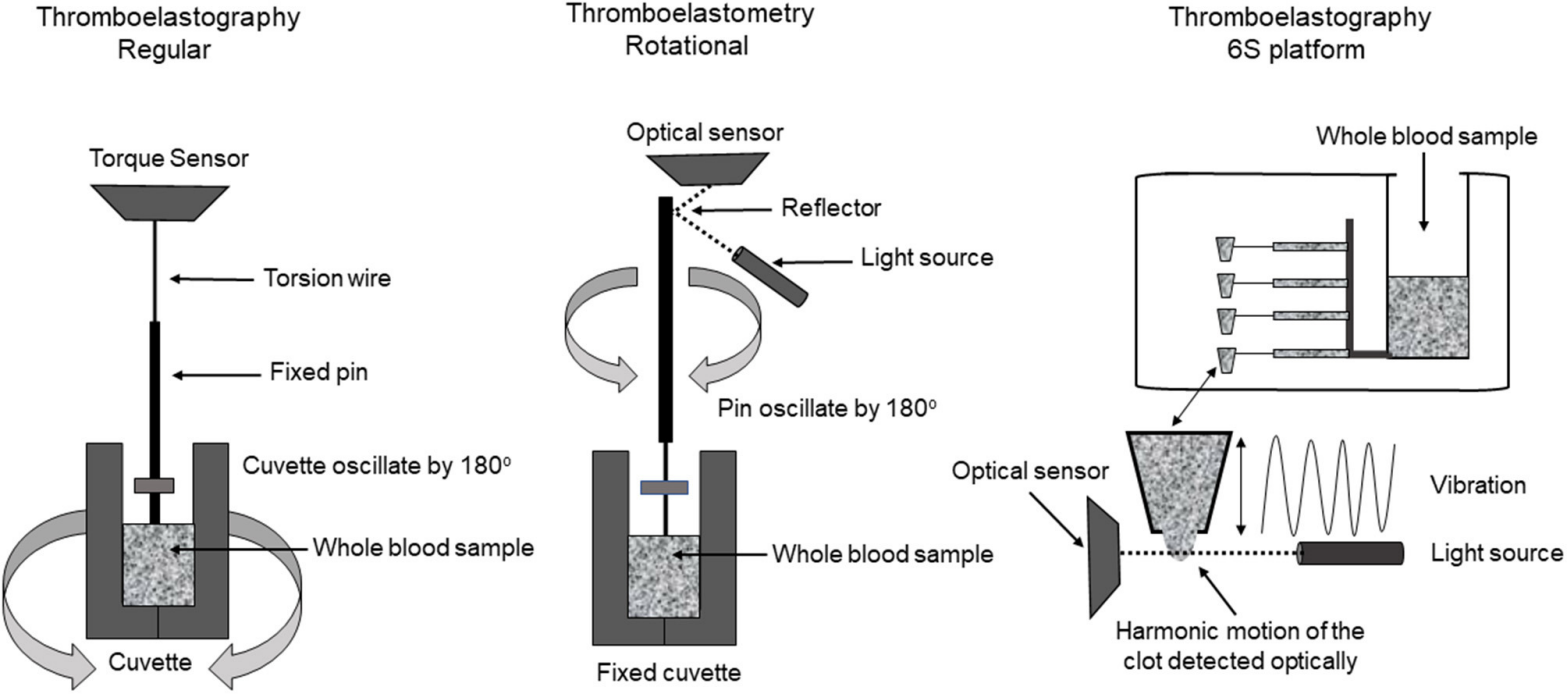
Viscoelastic testing laboratory techniques of TEG®5000, TEG®6S, and ROTEM®.

Today, there are two different technologies used to run TEG®, the TEG® 5000 and the TEG® 6S (Figure 2). The TEG® 5000 can analyze two samples simultaneously, using a fixed pin on a torsion wire suspended into an oscillating cylindrical cup containing a small amount of whole blood. The most commonly used activators include citrated kaolin, kaolin and tissue factor. As the first fibrin strands form and clot strength increases, more rotation is transmitted on the torsion wire, which is detected by an electromagnetic transducer (49, 50). The technique requires fine-tuned expertise for reproducible results but may also be adversely affected by the need for multiple blood sample transfers and vibration or movement of the machine.

The TEG® 6S was developed to overcome barriers seen with the TEG® 5000. TEG® 6S is an automated cartridge-based technology that uses a 4-channel microfluidic system containing the required reagents (citrated kaolin, citrated kaolin + heparinase, kaolin and kaolin + heparinase) and automatically aspirates citrated blood into the different test chambers (50). The system is compact with easy portability and requires only one blood sample transfer from the operator into the cartridge. In this test, as the clot formation and propagation occur, the clot is exposed to the high-frequency ultrasound pulses; with clot strength being measured by a resonance method (50).

Both systems have the ability to assess platelet function through the TEG® Platelet Mapping® (TEG®PM) (Haemonetics Corp, Braintree, MA, USA) assay. The assay assesses degree of platelet inhibition by comparison of the patient's full hemostatic potential and the difference when looking at the contribution of platelets through activation of specific receptors [e.g., adenosine diphosphate (ADP) and arachidonic acid (AA)].

Automated TEG® variables are described in Table 1. In addition to these parameters, several TEG® derived parameters can assist with overall thrombotic potential, including the clotting index (CI) and the shear modulus strength (G). The CI is a global assessment of clot formation that uses a linear combination of the R-time, K-time, MA and alpha angle. Using the amplitude at the MA variable, G is calculated in Kdynes/cm^2^ and represents maximum clot strength.

**Table 1:** Description of TEG® and ROTEM® parameters.

**Description and interpretation**	**TEG^®^ variable**	**ROTEM^®^ variable**
Duration from start of test until the clot reaches 2 mm amplitude; representative of coagulation factors	Reaction time (R)	Clotting time (CT)
Time it takes the amplitude to go from 2 to 20 mm; represents clot propagation	Kinetic time (K)	Clot formation time (CFT)
The slope between R or CT and K or CFT; represents rate of clot formation	Alpha angle (α-angle)	Alpha angle (α-angle)
Measurement of maximum clot strength; influenced by fibrinogen and platelet count	Maximum amplitude (MA)	Maximum clot firmness (MCF)
The difference between the MA or MCF and the amplitude of the curve after 30 min	Percent lysis at 30 min (Ly30)	Lysis index 30 (LI30)

#### ROTEM®

The thromboelastometry (ROTEM®) system utilizes a fixed cylindrical cup containing a small amount of whole blood while a pin oscillates through application of a constant force (Figure 2). As clot strength increases, the rotating pin is obstructed and is detected optically using a charge-coupled device image sensor system. A standard ROTEM® device can analyze four samples simultaneously and utilizes automated pipetting (49). The ROTEM® provides the same information as the TEG® but uses different terminology (Table 1). ROTEM® may also be reported based on the activator used (Table 2). It is important to note that although the same information is achieved, the results are not interchangeable as the mechanisms of each assay are different.

**Table 2:** Description of the ROTEM® tests available based on activator utilized.

**ROTEM^®^ test based on activator used**	**Description and interpretation**
INTEM	Contact activation; provides information similar to aPTT
EXTEM	Tissue factor activation; provides information similar to PT
HEPTEM	Contains heparinase to neutralize unfractionated heparin; compared with INTEM to assess heparin effect
APTEM	Contains aprotinin to inhibit fibrinolysis; compared with EXTEM to assess fibrinolysis
FIBTEM	Uses cytochalasin-D to block the platelet contribution to clot formation; compared with EXTEM to assess fibrinogen contribution to clot strength independent of platelets

### Interpretation in Clinical Practice

Most commonly, the samples obtained for VET are collected in sodium citrate tubes which necessitates timely processing. Standard values have been reported in healthy children and in children with congenital heart disease (51, 52). However, it has been reported that the use of samples collected in this way may result in significant artifacts on TEG® analysis and could lead to heparin overdosing in these patients (53). In addition, discrepant results have been reported using TEG® 5000, TEG® 6S and ROTEM® on the same whole blood sample in orthotopic liver transplant and trauma patients; although all three tests could identify abnormalities in clot formation (50, 54). If TEG® 6S provides an accurate assessment of coagulopathy in the ECMO and VAD population, its use at the bedside would allow for point of care results and decreased need for skilled laboratory personnel.

Another benefit of VET testing is the value in patients anticoagulated with DTIs. Dilute thrombin time is not readily available in many institutions and standard laboratory monitoring may not accurately reflect anticoagulant effect of DTIs. TEG® ecarin clotting time has shown excellent correlation with chromogenic bivalirudin levels, *r*^2^ = 0.746 (55). In a pediatric study including 106 blood samples of children supported with ECMO on bivalirudin, prolongation of clotting time on ROTEM intrinsic coagulation pathway (INTEM) and INTEM with heparinase (HEPTEM) showed moderate to strong correlation with aPTT and hepzyme aPTT suggesting that ROTEM may provide a good alternative to those standard assays. In this report, the target range of INTEM CT was 260–300 s for children without evidence of heparin-like activity anticoagulated with bivalirudin during ECMO (56).

Management of anticoagulation is predominantly assessed by using the R-time (TEG®) or CT (ROTEM®) and comparing the values with and without the presence of heparin. Reference ranges are based upon individual lab standardization. In an adult randomized controlled trial, anticoagulation using aPTT based protocol compared to TEG® based protocol targeting TEG®-Kaolin R-time 16–24 min (normal value: 4–8 min) found that TEG based titration was safe, feasible, and resulted in lower heparin dose compared to aPTT based protocol (57). A pediatric retrospective study found that anti-FXa activity > 0.25 IU/ml (sensitivity 81%, specificity 67%, PPV 81%, and NPV 58%) and a TEG® R-time > 17.85 min (sensitivity 84%, specificity 68%, PPV 82%, and NPV 59%) may minimize the risk of thrombosis in pediatric and neonatal ECMO patients but could not find an optimal target to minimize bleeding (58). In general, programs target somewhere between 2 and 4 times the baseline TEG® R-time. In the event of incomplete correction of the R/CT time with the addition of heparinase, it may suggest coagulation factors deficiency and consideration may be given to replacement with FFP based on presence or absence of clinical bleeding. In addition, some patients develop acquired von Willebrand deficiency or other clotting factor deficiencies due to consumption in the circuit and abnormalities can prompt additional laboratory testing to identify deficiencies. Clot strength as assessed by the MA/MCF is influenced by fibrinogen, absolute platelet count and function. Increased lysis time may be indicative of hyperfibrinolysis (circuit disseminated intravascular coagulopathy or acquired factor XIII deficiency). Importantly, viscoelastic tests may help diagnose covert coagulopathy that may not be identified on standard laboratory assays and could contribute to bleeding risk (59).

### Evidence to Support VET Use in ECMO and VAD

**Figure 3:**
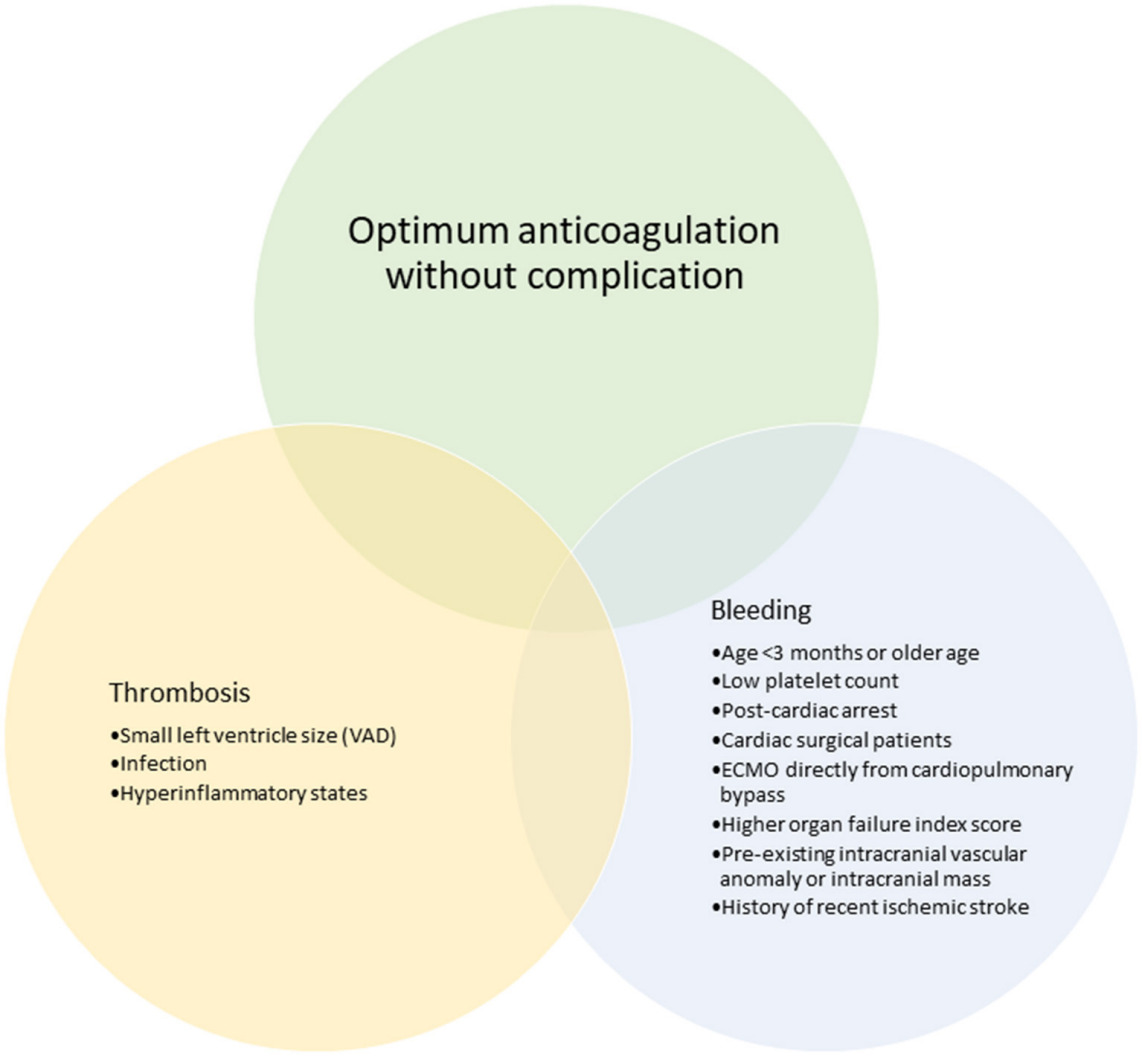
Clinical features that have been associated with bleeding and thrombotic complications in pediatric ECMO and VAD.

**Figure 4:**
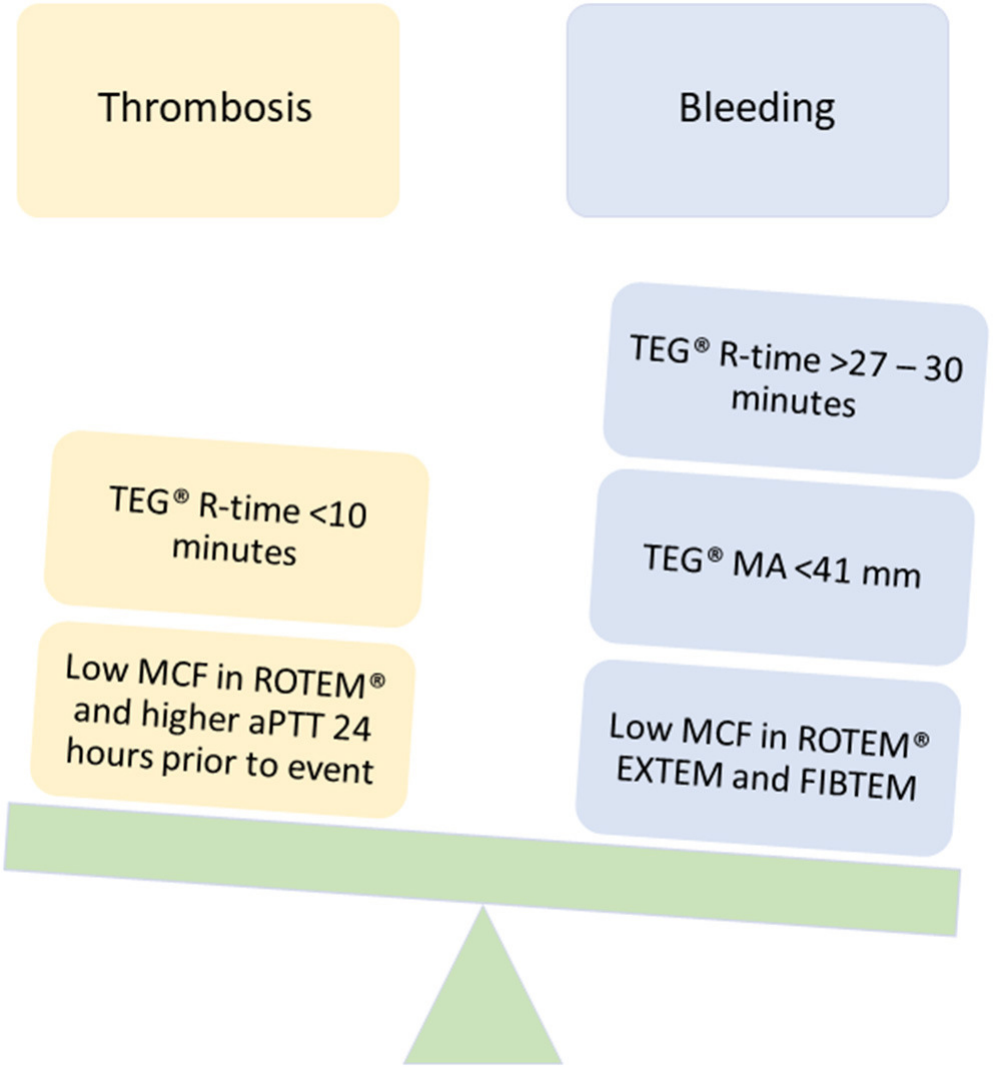
TEG®/ROTEM® parameters that have been associated with bleeding and thrombotic complications in pediatric ECMO and VAD.

Although limited, studies evaluating the use of VET in adult ECMO and VAD have increased over the last decade. Pediatric ECMO/VAD studies have reported VET-based anticoagulation algorithms and associations with clinical outcomes including bleeding and thrombosis. Figures 3, 4 describe the clinical features and TEG®/ROTEM® parameters that have been associated with increased bleeding and thrombotic complications. In a recent report using data from 265 children in the Pediatric ECMO Outcomes Registry (PEDECOR) VET was utilized in $\frac{3}{4}$ of centers contributing data, although bleeding and thrombotic complications were not different between centers utilizing VET compared to centers that did not (60). We review these pediatric studies below and in Table 3.

**Table 3:** Pediatric ECMO and VAD studies that have analyzed the use of VET.

**Study (Paper)**	**Study design**	**Location**	**Years**	**# Pediatric patients (ECMO/VAD)**	**TEG^®^ vs.** **ROTEM^®^**	**Important findings**
Stammers (61)	Retrospective NRCT	USA	1995	5–VV ECMO 12–VA ECMO	TEG®	•TEG® showed abnormal profile in 17% of patients without hemorrhagic complications compared to 60% of patients with hemorrhagic complications (*p* < 0.001). •TEG® provides useful information for rapid diagnosis of hemorrhagic conditions, which may help guide transfusion therapy.
Alexander (62)	Retrospective NRCT	Australia	2006–2008	1–VV ECMO 20–VA ECMO 3–VAD 3–ECMO + VAD	TEG®	•TEG® heparinase samples (including R-time, K-time, α-angle, and R+K) had a weak correlation to aPTT and ACT.•A stronger correlation was seen with TEG® heparinase (MA) and platelet count (*r* = −0.4, *p* < 0.001).
Davis (63)	Case report	Denmark	2006	1–VA ECMO	TEG®	•TEG® used to monitor use of Novo7 in a case of major coagulopathy.
Northrop (64)	Retrospective NRCT	USA	2007–2013	366–ECMO	TEG®	•Use of a comprehensive ECMO anticoagulation laboratory protocol, including anti-FXa, TEG® and AT measurements is associated with decreased blood product administration, decreased hemorrhagic complications and increased ECMO circuit life.
Laine (65)	Prospective NRCT	Finland	2008–2012	5–VV ECMO 18–VA ECMO 8–BiVAD 16–LVAD	ROTEM®	•Low MCF in EXTEM and FIBTEM was associated with severe bleeding.•Low MCF in FIBTEM was associated with 30-day mortality.
Phillips (66)	Retrospective NRCT	USA	2008–2018	46–ECMO	TEG®	•Two groups evaluated, pre-2015 (*n* = 24) and post-2015 (*n* = 22), after implementation of standardized anticoagulation protocol that incorporated TEG®.•After addition of TEG® technology, there was an increase in platelet transfusion for low MA. However, cryoprecipitate was given less frequently for low α-angle.•After addition of TEG® technology, significant reduction in hemothoraces (18% compared to 54%, *p* = 0.026).
Ranucci (67)	Retrospective NRCT	Italy	2008–2015	31–VA ECMO (post-cardiotomy) (*n* = 10 pediatric patients, *n* = 21 adults)	TEG®	•Strong predictive value for ACT <162 s and R-time <10 min for a short aPTT.•Moderate predictive value for ACT >185 s with an R-time >27 min for a long aPTT.•Moderate predictive value for MA <41 mm for a low platelet or fibrinogen count.
Giorni (35)	Retrospective NRCT	France	2009–2014	6–BH, BiVAD 1–BH, LVAD	TEG®PM	•Antiplatelet therapy monitoring in BH-implanted children remains challenging; the TEG®PM is sensitive to many preanalytical and analytical conditions.
Rosenthal (24)	Retrospective NRCT	USA	2009–2014	10–BH, BiVAD 17–BH, LVAD	TEG®PM	•Assessed two cohorts, using Edmonton Anti-thrombotic Guideline and TEG®PM (EG) to guide dual antiplatelet therapy vs. Stanford Modified Anti-thrombotic Guideline (SG) with higher weight-based dosing for triple antiplatelet therapy.•Incidence of stroke and bleeding rate was lower in SG cohort.•No difference in ADP inhibition by TEG®PM, but AA inhibition was higher in SG cohort (median 75 vs. 39%, *p* = 0.008).
Burton (60)	Retrospective NRCT	USA	2011–2018	98–VV ECMO 375–VA ECMO	TEG® and ROTEM®	•Platelet dysfunction was the most common abnormality identified by TEG® and ROTEM®.•Cryoprecipitate was utilized more often in bleeding patients who had VET performed than those who did not have VET.•Median R-time for TEG® without heparinase was 23.5 min and median ROTEM® INTEM CT was 224.1 s, which may be potential targets for UFH dosing in pediatric ECMO.
Drop (68)	Retrospective NRCT	Netherlands	2011–2018	30–VV ECMO 43–VA ECMO	ROTEM®	•Low MCF in all ROTEM® components and a higher minimum aPTT 24 h prior to an event were associated with increased thrombotic risk.•ROTEM® CT was not associated with thrombotic events.•No association of bleeding complications and results of ROTEM®, aPTT and anti-FXa.
Saini (69)	Retrospective NRCT	USA	2011–2012	18–VV ECMO 6–VA ECMO	TEG® + Multiplate platelet aggregometry	•Using TEG®PM, severe qualitative platelet dysfunction was more common for ADP (92%) compared to AA (75%) [*p* = 0.001].•Absolute platelet count and TEG®PM had increased predictive value for severe bleeding and mortality compared to ACT.•No difference in kaolin-activated heparinase TEG® parameters between bleeding and non-bleeding group.
Bhatia (70)	Retrospective NRCT	USA	2013	4–BH, LVAD	TEG®	•Demonstrated association between aPTT and TEG® R-time (Rs = 0.65, *p* < 0.001) and between anti-FXa and TEG® R-time (Rs = 0.54, *p* < 0.001).•Significant correlation between platelet counts and TEG® MA (Rs = 0.71, *p* < 0.001).•Despite correlation of monitoring assays, there is a lack of correlation between heparin dose and degree of effect.
Bingham (71)	Retrospective NRCT	USA	2013–2016	35–ECMO	TEG®	•TEG® R-time <30 min significantly decreased amount of major bleeding, AUC 0.76 (*p* < 0.001).
Henderson (58)	Retrospective NRCT	USA	2013–2015	4–VV ECMO 26–VA ECMO	TEG®	Anti-FXa (OR = 0.62) and TEG® R-time (OR = 1.19) were independent predictors for significant thrombotic events.•Targeting an anti-FXa activity >0.25 IU/ML and TEG® R-time >17.85 min may minimize risk of thrombosis in pediatric and neonatal ECMO patients.
Ferguson (72)	Retrospective NRCT	United Kingdom	2015–2016	5–BH, BiVAD 3–HeartWare HVAD 1–Thoratec CentriMag	TEG®PM	•Rate of aspirin and clopidogrel resistance was higher in TEG®PM than multiplate electrode platelet aggregometry.•Multiple electrode platelet aggregometry was more reliable than TEG®PM for monitoring antiplatelet therapy in pediatric patients supported with VAD.
Moynihan (73)	Retrospective NRCT	Australia	2015–2016	5–VV ECMO^*^ 29–VA ECMO	TEG®	•Anti-FXa and TEG®6S showed best correlation with heparin dose, although association was low.•Low anti-FXa was observed in ECLS runs with thrombotic complications.
Teruya (56)	Retrospective NRCT	USA	2017–2018	6–ECMO 12–VAD	ROTEM®	•Strong correlation between HaPTT and HEPTEM CT, and a moderate correlation between aPTT and INTEM CT.•Strong correlation between FIBTEM MCF and fibrinogen levels.•FIBTEM overestimated fibrinogen level when platelet count was >300 k.
Rabinowitz (74)	Retrospective NRCT	USA	2018–2020	21–VV ECMO 43–VA ECMO 1–VAV ECMO 2–VVA ECMO	TEG®	•No consistent correlation between anticoagulant dosing and at least one laboratory parameter.•Inconsistencies in the correlation of anticoagulation dosing and laboratory parameters highlight the importance of multimodal management model.
Yabrodi (59)	Retrospective NRCT	USA	2020	100–VA ECMO	TEG®	•Both heparin dose and anti-FXa levels showed a low, but statistically significant correlation with TEG R-time.•Monitoring with heparinase TEG® may be helpful to diagnose coagulopathy in ECMO.

* *These five patients are a part of the 29 VA ECMO patients who were transitioned to VV. NRCT, nonrandomized controlled trial; ECMO, extracorporeal membrane oxygenation; anti-FXa, anti-factor Xa; AT, antithrombin; VV, venovenous; VA, venoarterial; BH, Berlin Heart; BiVAD, biventricular assist device; LVAD, left ventricular assist device; HaPTT, heparinase aPTT*.

#### VET Impact on Bleeding

Bleeding is the most commonly encountered hemostatic complication during ECMO/VAD. Adult studies of VET to predict and prevent bleeding have reported mixed results. Panigada et al. randomized patients to a TEG®-based protocol vs. a standard of care aPTT-based protocol and found more anticoagulation dosing adjustments were made in the TEG® group, lower dose of heparin was administered, and target parameters were less frequently in therapeutic range in the TEG® group. Despite these findings, there were no significant differences in hemostatic complications or transfusion support (57). Conversely, a recent retrospective cohort study in adult ECMO patients compared pre-protocol (aPTT alone, goal 60–80 s) to post-protocol (aPTT, goal 60–80 s + TEG®, goal R-time 2–4 times baseline) and reported that overall bleeding complications were similar, but that there was a significant decrease in retroperitoneal bleeding events and that overall mortality during ECMO support was significantly lower in the post-protocol group (75).

In a meta-analysis on the impact of point-of-care testing in adult and pediatric ECMO patients, the use of VET was associated with lower in-hospital mortality and decreased bleeding events, but this was not statistically significant. There was a significant reduction in the frequency of surgical revision for bleeding (76). Hypocoagulability and bleeding complications have been correlated with ROTEM® in both ECMO/VAD. A prospective study including 24 ECMO and 23 VAD adult patients found that a decreased maximum clot firmness (MCF) in EXTEM and FIBTEM was associated with severe bleeding, and decreased MCF in FIBTEM was associated with increased 30-day mortality risk (65).

Clinical data supporting VET specific to the neonatal and pediatric population are limited to a few prospective studies. In pediatric ECMO, most reports are retrospective chart reviews utilizing TEG® and TEG®PM. In CDH neonates, the addition of TEG® for monitoring showed a significant reduction in hemothoraces and improved blood product utilization (66). However, correlation of the TEG® parameters to other monitoring tests (ACT and aPTT) are challenging due to the variability of heparin effect. Alexander et al. found a weak correlation of the TEG® heparinase variables and the aPTT/ACT, but a stronger correlation between the TEG® MA and platelet count (62). In the PEDECOR study, VET identified platelet dysfunction most commonly and bleeding patients who had VET performed received more cryoprecipitate transfusions than those who did not have VET (60). A chart review of children <18 years on ECMO looked at severe platelet dysfunction (<50% aggregation on TEG®PM) and bleeding. Severe platelet dysfunction was more common for ADP (92%) vs. arachidonic acid (75%) but no difference in the TEG® parameters between the bleeding and non-bleeding group. However, the absolute platelet count and TEG®PM had increased predictive value for severe bleeding and mortality compared to monitoring with ACT (69). Although TEG®PM is used less frequently in ECMO, this study suggests it may aid in prevention of severe bleeding. Importantly, TEG® has been reported to aid in detection and management of hyperfibrinolysis (77). Similar findings have been reported with the use of ROTEM® in pediatrics. A retrospective observational cohort study, including 73 pediatric patients found no association between bleeding complications and results of the ROTEM®, aPTT, and anti-FXa. However, the multivariate analysis showed that surgical intervention and age <29 days significantly increased the incidence of major bleeding events (68). Conversely, as mentioned above, Teruya et al. found that the ROTEM® CT on INTEM and HEPTEM had a moderate to strong correlation with the aPTT and HaPTT for children receiving bivalirudin for anticoagulation which may be a major application of VET for pediatric ECMO patients (56).

#### VET Impact on Thrombosis

Thrombotic complications can range from mild to severe and have detrimental effects on patient outcomes. Conventional monitoring tests are associated with significant variability and have not been shown to reliably correlate with thrombosis. Unfortunately, to date the correlation of VET with thrombosis has also been limited (65, 76). A recent retrospective pediatric ECMO cohort study reported reduced MCF (in all ROTEM® components) and higher minimum aPTT 24 h was associated with increased thrombotic risk (68). While limited, this study suggests that ROTEM® could be more predictive of thrombotic risk than aPTT and in this scenario could aid clinicians in assessment of thrombotic risk and reevaluation of targeted management/prevention.

VET has been applied more frequently in the pediatric VAD population. Dosing of dipyridamole and aspirin were found to increase across all ages with increasing duration of support, which may indicate progressive inflammation, vasculopathy or adverse thrombotic events (3). TEG® or ROTEM® with platelet mapping has been used to identify inadequate platelet inhibition and its routine use may allow prevention of thombotic complications (78). Unfortunately, the clinical utility of TEG®PM has been questioned due to technical challenges resulting in variable test results and validity must be confirmed by visual interpretation of the generated curve within certain criteria (79). This makes the application of TEG®PM limited in universal application and more reliant on experienced specialist interpretation that may not be available at all centers. Despite these limitations, in the ACTION DTI harmonization protocol, aspirin effect is monitored either by TEG®PM or the functional assay VerifyNow which evaluates platelet aggregation by a turbidimetric-based optical detection (80).

#### VET Impact During COVID-19

Severe COVID-19 respiratory illness has a significant impact on hemostatic balance and the systemic inflammation seen in these patients contributes to a hypercoagulable state. Yuriditsky et al. reported that in severe COVID-19 illness, the TEG® was able to reliably identify this hypercoagulable state. The alpha angle and MA were elevated in 70 and 60%, respectively. The clotting index was hypercoagulable (>3) in 50% of patients with severe COVID-19 and 31% of these patients suffered from thromboembolic events. The correlations between TEG® and traditional inflammatory/coagulation laboratory values was weak to moderate suggesting that TEG® was better able to predict thromboembolic event then routine laboratory tests (81). Subtle changes in TEG® or ROTEM® variables may precede changes in routine laboratory values (i.e., fibrinogen) and clinical picture (i.e., hyperfibrinolysis) before a major hemostatic event occurs (82). Specifically, the use of VET during COVID-19 adult ECMO has found associations with d-dimer, fibrinogen and TEG® MA values and arrest of fibrinolysis as demonstrated by the TEG® Ly30 (20, 83, 84).

In pediatric patients with acute COVID-19 infection, one small case series reported ROTEM® parameters associated with hypercoagulable state but no clinically significant thrombotic events (85). In pediatric MIS-C, a single center report (*n* = 30) showed patients had evidence of hypercoagulability on TEG® but similarly did not demonstrate any symptomatic thrombotic event or major bleeding (86). In a single center cohort (*n* = 22) of pediatric patients with acute COVID-19 and MIS-C, TEG® uncovered a pattern of accelerated clot formation and increased clot strength in a subset of children and unlike the previous reports this cohort included two patients with significant thrombotic complications (87).

### Limitations of VET in Pediatric ECMO/VAD

While the use of VET in pediatric ECMO/VAD has increased, limitations exist. Importantly, technical considerations, including skills to properly maintain the device and expert proficiency in handling blood samples, are needed for reliable results. After sample procurement, activation of the sample is performed. In TEG® and ROTEM, contact activation (*intrinsic coagulation pathway*) using ellagic acid or kaolin can provide information like activated partial thromboplastin time. While tissue factor activation (*extrinsic coagulation pathway*) provides information like prothrombin time. Lyophilized heparinase neutralization of heparin, blocking of platelet contribution to clot formation with platelet inhibitors, or inhibiting fibrinolysis with antifibrinolytics can be used. Thus, activators for both the intrinsic and extrinsic systems are available. When celite and kaolin are used as activators for TEG® in the presence of aprotinin, there is a prolongation of R time, a decrease in α angle, and a decrease in MA, suggesting that these are not ideal activators for a TEG®-guided transfusion algorithm (88). TEG® has a poor correlation with frequently used laboratory testing and has not been shown to predict bleeding or thrombosis during ECMO. Currently, the cut-off values based on TEG® and ROTEM-based parameters are not well established for pediatric patients. In addition, results should be interpreted by an experienced clinician who understands the intricacies of the test and variables affecting the hemostatic system. Today, the amount of blood required to run the VET is small. Still, it may be problematic in the neonatal population, especially those awaiting cardiac transplantation, where it is essential to limit blood product exposure. Another limitation is that von Willebrand deficiency cannot reliably be detected using VETs. In addition to all of these factors, the child's clinical status is the most critical variable, and decisions based on VET/laboratory tests may not reflect the dynamic changes in the critically ill child.

## Future Considerations

With clot development, the state of blood changes from a viscous fluid to a semisolid gel, and these changes can be detected by sonic estimation of elasticity *via* resonance (SEER) sonorheometry (Quantra™). This newer ultrasound-based VET uses high frequency ultrasound pulses to characterize the dynamic changes in viscoelastic properties of a blood sample during coagulation. Clot stiffness as measured by SEER have shown strong positive correlations in TEG® and ROTEM® looking at G and FIBTEM, respectively; as well as a strong correlation to fibrinogen and platelet count (89, 90).

Another area under investigation is the use of a shear gradient activated microfluidic approach. The microfluidic device mimics stenosed arteriolar vessels, allowing for the assessment of thrombotic potential within small sample volumes under pathophysiologic flow. This technology has shown when integrated into the extracorporeal circuit in pig endotoxemia or heparin therapy models, it reports real time data of alterations in coagulation *ex vivo* that are more reliable than standard coagulation assays (e.g., ACT and aPTT) (91). The introduction of this technology into human mechanical circuits could kickstart the possibility of personalized diagnostics and minute-to-minute real-time surveillance of the hemostatic balance and reduce need for blood draws. Unfortunately, this technology is currently available only for research studies or in limited academic institutions.

## Conclusions

The use of VET for guiding anticoagulation management in pediatric ECMO and VAD is increasing. Studies are limited in this population and no high quality data supports VET algorithms over standard coagulation testing. However, VETs may provide benefit in detecting subtle changes in this high-risk population. Incorporation of these assays into general monitoring practice may aid in precision anticoagulant management. VET use in COVID-19 is promising. Additional studies are needed in the pediatric ECMO and VAD population to determine optimal role of VET.

## Author Contributions

KR wrote the manuscript draft and finalized changes after revision and approval. AS and KC revised, read and approved final manuscript.

## Conflict of Interest

The authors declare that the research was conducted in the absence of any commercial or financial relationships that could be construed as a potential conflict of interest. The reviewer HJD declared a past co-authorship with the author KC to the handling editor.

## Publisher's Note

All claims expressed in this article are solely those of the authors and do not necessarily represent those of their affiliated organizations, or those of the publisher, the editors and the reviewers. Any product that may be evaluated in this article, or claim that may be made by its manufacturer, is not guaranteed or endorsed by the publisher.

## Abbreviations

AA: Arachidonic acid
ACT: Activated clotting time
ACTION: Advanced cardiac therapies improving outcome network
ADP: Adenosine diphosphate
Anti-FXa: Anti-factor Xa
aPTT: Activated partial thromboplastin time
AT: Antithrombin
CT: Clotting time
DTI: Direct thrombin inhibitor
DTT: Dilute thrombin time
ECMO: Extracorporeal membrane oxygenation
ECPR: Extracorporeal cardiopulmonary resuscitation
EXTEM: Extrinsic thromboelastometry
FFP: Fresh frozen plasma
FIBTEM: Functional fibrinogen thromboelastometry
FXa: Factor Xa
HaPTT: Heparinase activated partial thromboplastin time
HEPTEM: Heparinase thromboelastometry
HIT: Heparin induced thrombocytopenia
INTEM: Intrinsic thromboelastometry
K-time: Kinetic time
Ly30: Clot lysis time
MA: Maximum amplitude
MCF: Maximum clot firmness
MIS-C: Multisystem inflammatory syndrome in children
R-time: Reaction time
ROTEM®: Thromboelastometry
TEG®: Thromboelastography
TEG®PM: Thromboelastography with platelet mapping
UFH: Unfractionated heparin
VA: Venoarterial
VAD: Ventricular assist device
VET: Viscoelastic testing
VV: Venovenous
vWF: von Willebrand factor.
